# Acceptability and feasibility of video-based coaching to enhance clinicians’ communication skills with patients

**DOI:** 10.1186/s12909-021-02976-2

**Published:** 2022-02-08

**Authors:** Jennifer Freytag, Jinna Chu, Sylvia J. Hysong, Richard L. Street, Christine M. Markham, Thomas P. Giordano, Robert A. Westbrook, Sarah Njue-Marendes, Syundai R. Johnson, Bich N. Dang

**Affiliations:** 1 VA Center for Innovations in Quality, Effectiveness, and Safety, Houston, USA; 2 Michael E. DeBakey VA Medical Center, Houston, USA; 3 Baylor College of Medicine, Houston, USA; 4 Texas A&M University, College Station, USA; 5 University of Texas Health Science Center, Houston, USA; 6 Jesse H. Jones Graduate School of Business, Rice University, Houston, USA

**Keywords:** Patient-clinician communication, Feedback, Video coaching

## Abstract

**Background:**

Despite a growing call to train clinicians in interpersonal communication skills, communication training is either not offered or is minimally effective, if at all. A critical need exists to develop new ways of teaching communication skills that are effective and mindful of clinician time pressures. We propose a program that includes real-time observation and video-based coaching to teach clinician communication skills. In this study, we assess acceptability and feasibility of the program using clinician interviews and surveys.

**Methods:**

The video-based coaching intervention targets five patient-centered communication behaviors. It uses trained communication coaches and live feed technology to provide coaching that is brief (less than 15 min), timely (same day) and theory-informed. Two coaches were trained to set up webcams and observe live video feeds of clinician visits in rooms nearby. As coaches watched and recorded the visit, they time stamped illustrative clips in real time. Video clips were a critical element of the program. During feedback sessions, coaches used video clips to promote discussion and self-reflection. They also used role play and guided practice techniques to enforce new tips. Clinicians included residents (*n* = 15), fellows (*n* = 4), attending physicians (*n* = 3), and a nurse practitioner (*n* = 1) at two primary care clinics in Houston, Texas. We administered surveys to clinicians participating in the program. The survey included questions on quality and delivery of feedback, and credibility of the coaches. We also interviewed clinicians following the intervention. We used rapid analysis to identify themes within the interviews.

**Results:**

Survey measures showed high feasibility and acceptability ratings from clinicians, with mean item scores ranging from 6.4 to 6.8 out of 7 points. Qualitative analysis revealed that clinicians found that 1) coaches were credible and supportive, 2) feedback was useful, 3) video-clips allowed for self-reflection, 4) getting feedback on the same day was useful, and 5) use of real patients preferred over standardized patients.

**Conclusions:**

Video-based coaching can help clinicians learn new communication skills in a way that is clinician-centered, brief and timely. Our study demonstrates that real-time coaching using live feed and video technology is an acceptable and feasible way of teaching communication skills.

## Contributions to the literature

- We describe the development of a video-based communications coaching program that is brief, same day, and well-received by clinicians.
- We present a feasible task-oriented way to teach clinicians specific and concrete communication skills.
- We show that training non-clinician coaches to conduct real-time video coaching sessions with clinicians is feasible and provide guidance for future implementation of this approach.

## Background

Effective communication is of critical importance in developing relationships between patients and clinicians. Positive patient-clinician relationships impact health outcomes across a variety of medical conditions and care settings [1]. Patients with positive relationships have greater trust in their clinician and are more likely to take their medicines and engage in care [2–5]. Positive relationships and effective communication also have the potential to mitigate racial and ethnic disparities in healthcare. Effective communication may have stronger effects on trust and feeling respected by the clinician in African American and Hispanic patients [6–9].

Despite the importance of effective clinician communication, teaching clinicians complex communication skills is difficult, and few evidence-based interventions exist. Most interventions focus on workshops and traditional didactic training, with largely mixed results. Some studies have found a weak correlation between these interventions and improved communication behaviors [10–13]; others have found no correlation [14–19]. Given mixed results, a “basic science” of communication behaviors that can be taught and measured must be established. This requires breaking down communication into concrete elements that drive effective communication and determining the best ways to teach these elements to clinicians. These data are key to developing targeted and effective interventions.

In the United States, the Accreditation Council for Graduate Medical Education (ACGME) cites interpersonal and communication skills as core clinical competencies [20]. However, most medical schools lack the expertise to teach communication skills effectively [21, 22]. Medical students interviewed about their training in communication report that they are rarely observed, and feedback, if any, is not timely [23]. Most clinicians ultimately finish their medical education with little training in communication skills [23–25]. When training occurs after residency, it typically takes place in workshops that may or may not include simulated patient encounters. These workshops are time-intensive and difficult for clinicians in busy clinical environments to attend [21]. Most workshops take place as half to all day one-time events, and little data exists on the long-term effects of these training [26]. Given these challenges, a critical need exists to develop new ways of teaching communication that are effective and mindful of clinician time pressures. We propose that a program with real-time coaching, using live feed and video technology, meets the challenge of teaching communication skills. In this study, we use qualitative and quantitative methods to examine the acceptability and feasibility of a real-time video-based coaching program delivered to clinicians in busy primary care clinics at a Veterans Affairs (VA) hospital and a county clinic.

### Communication behaviors

A crucial step in developing clinicians’ communication skills is identifying specific, evidence-based communication behaviors that promote positive patient-clinician relationships. Patient-centered communication, considered the gold standard of patient-clinician communication, includes (1) explicitly asking if patients have questions (2), giving clear information about the patient’s condition (3), focusing on objective measures (4), involving patients in the conversation, and (5) acknowledging patients' emotions [27].

#### Explicitly asking if patients have questions

When patients ask questions during clinic visits, they engage in conversation and prompt clinicians to provide answers [28]. However, patients often feel they should not ask questions for fear of being labelled ‘difficult.’ [29] Patient reticence to ask questions can be addressed by teaching clinicians to explicitly ask patients if they have questions (e.g. “What questions do you have today?”) [27].

#### Giving clear information about the patient’s condition

Giving medical information in a way that is clear increases patient understanding and promotes a positive patient-clinician relationship. Previous work has shown that patients value clear information about their treatment plan [27]. It is also particularly important for clinicians to support their recommendations with a rationale, to provide information in small chunks, and to check for patient understanding [30]. When possible, clinicians should also share with patients the specific, objective measures that are the basis of their recommendations, such as blood pressure or cholesterol readings, particularly when the conversation involves a condition that might make patients feel judged, such as overweight.

#### Involving patients in the conversation

Eliciting patient input aids in the development of positive patient-clinician relationships. Directly asking patients how they feel about a treatment option or what they think is causing their symptoms, for example, provides patients with support and further builds patient-clinician partnerships [31].

#### Acknowledging patients' emotions

The same can be said for acknowledging patients' emotions. Clinicians often find responding to patient emotions hard, and particularly feel less in control of the patient encounter when patients express negative emotions [32]. However, acknowledging when a patient expresses emotion strengthens the patient-clinician relationship.

### Audit and feedback

Audit and feedback is an evidence-based teaching method for improving performance. The method derives from Industrial/Organizational psychology (where it is known as ‘feedback’) and is used widely across diverse industries (e.g., education, athletics, aerospace). In healthcare, audit and feedback interventions have decreased the inappropriate use of antibiotics, reduced unnecessary imaging, and educated patients to manage their health conditions [33–35]. Feedback Intervention Theory (FIT) posits that feedback works by increasing awareness of a gap between a desired behavior and a person’s actual behavior, thus prompting change [36]. Per FIT, three key factors determine the effectiveness of feedback interventions. These include feedback cues directed at changing behavior, the task at hand, and situational factors (e.g. feedback orientation, sense of control, and threats to self-esteem) [37–39]. To maximize effectiveness, feedback cues should include information on the correct solution (i.e., tell/show the recipient “what ‘good’ looks like”) [40] and be delivered in a timely, individualized, non-punitive, and ideally customizable fashion [41].

According to FIT, an effective audit and feedback intervention needs to target specific tasks or steps critical to improving performance. In communication, this requires breaking down broad communication goals into critical elements. For example, clinician actions to increase patient involvement would be broken down into concrete communication tasks. These could include tasks such as using open-ended prompts (“Tell me about your condition”) or asking the patient to list goals for the visit (“What would you like to talk about today?”). Breaking down broad performance goals into key individual components, helps reframe communication as a set of tasks at the right level of specificity for delivering actionable, evidence-based feedback. This in turn allows clinicians to focus on specific, actionable communication skills that can lead to improved communication performance as a whole [42].

A challenge in providing feedback on communication skills is the personal and idiosyncratic nature of communication behavior [43]. Communication tasks are inherently interwoven in complex patterns of learned behavior (i.e., the long-established communication habits we use everyday) [44]. This suggests that more so than other practices, feedback on clinician communication requires performance evaluations that focus on specific behaviors in a manner that does not threaten a person’s individual communication “style” or habits. The manner in which feedback is delivered matters, and care needs to be taken to create a safe environment in providing and receiving feedback [44, 45].

## Methods

### Participants

Recruitment for clinicians and patients took place at two primary care clinics in Houston, Texas, between October 2018 and October 2019 (Prime Care clinic at the Michael E. DeBakey VA Medical Center, and Thomas Street Health Center, a free-standing primary care clinic for patients with HIV infection). The Principal Investigator (Dang) developed relationships with site “champions,” leaders at both clinics who supported the coaching intervention and introduced the PI and members of the research team to potential participants at clinician meetings (e.g. morning report, noon conference, journal club). Residents at the VA were offered a half-day of independent study, and all other participants were offered $20 for participation in the coaching intervention. Members of the research team obtained consent from both participating clinicians and patients recorded during visits. Both pilots were approved by Baylor College of Medicine’s Institutional Review Board, and the VA pilot was also given permission by the Houston VA’s research review committee.

### Measures

This study takes two methodological approaches to assess feasibility and acceptability of a real-time video-based coaching intervention. We conducted a short survey with participating clinicians. Survey data included questions about the intervention’s acceptability, including the quality and delivery of feedback, as well as the credibility of the coaches. Questions were adapted from validated items used in Industrial/Organizational psychology to assess feedback environment [46–48]. We also interviewed clinicians to gather data on expectations and experiences with the program, and suggestions for improving the program. See Table 1 for key interview questions. Interviews with clinicians were audio recorded and professionally transcribed verbatim. Interviews ranged from 15 to over 20 min in length.

**Table 1:** Schedule of Interview Questions

**Clinician Questions**	
**Initial Prompt** I’d like to know what you think about the coaching and the feedback you received, and what we can do to improve the program. **Interest and Expectations** First, let’s talk about your interest in and expectations for the study. 1. Why did you decide to take part in the coaching program? 2. What expectations, if any, did you have when you decided to participate? **Coaching and Feedback** Now I’d like to talk about the coaching and the feedback you received. 3. What was it like being coached? PROBES: a. What did you like about the coaching? b. What did you not like about the coaching? 4. What did you think of the feedback the coach gave you? PROBES: a. Tell me about the feedback you got. b. How helpful or useful was the feedback? c. What was not helpful or useful? 5. What, if anything, do you plan to do differently based on the feedback you received? **Improving the Coaching Program** Now I’d like to talk about the coaching program in general. 6. What can we do to improve the coaching program? 7. Did any part of the coaching program negatively impact your work? PROBES: a. Clinic flow b. Rapport with your patient c. Clinic space d. Length of the coaching session e. Perceived burden f. Opportunity cost 8. What is the best way to integrate the coaching program into < VA/ TSC >?	

### Data analysis and rigor

We used descriptive statistics to analyze survey data and rapid qualitative analysis to analyze interview data. Rapid analysis is a practical method used often in health services and implementation science research [49, 50]. The goal is timeliness – to analyze data in a timely way and disseminate new ideas quickly. To conduct rapid analysis, two members of the research team (Njue-Marendes, Chu) independently created matrices, organizing quotes by interview questions (See Table 2). Matrices allow for quick comparison of interview data across participants, so that responses for each interview question can be easily examined for patterns and themes [51]. Responses were compared, discussed, and reexamined using a systematic and iterative process to identify final themes with exemplar quotations [52].

**Table 2:** Rapid Analysis Matrix

Theme	Interview question	Clinician 1	Clinician 2	….	Clinician 23
Interest and expectations	Why did you decide to take part in the coaching program?				
	What, if any, expectations did you have when you decided to participate?				
The coach	What was it like being coached?				
	What did you like about the coaching?				
	What did you not like about the coaching?				
Feedback received	What did you think of the feedback the coach gave you?				
	What, if anything, do you plan to do differently based on the feedback you received?				
The coaching program	What can we do to improve the coaching program?				
	Did any part of the coaching program negatively impact your work?				
	What is the best way to integrate the coaching program into your clinic?				

#### Description of the coaching intervention

The coaching program targets five explicit, clearly defined clinician communication behaviors that have potential to greatly improve the patient care experience [27]. It uses trained communication coaches and live feed technology to provide coaching that is brief (less than 15 min), timely (same day) and theory-informed. The coaching intervention uses live feeds to directly observe clinician communication behavior during a patient encounter. Live feeds let the coach observe communication behaviors in real time and provide timely feedback to the clinician. A coach trained in identifying specific clinician communication behaviors places an encrypted laptop with a webcam in the examination room before the patient-clinician encounter.

**Fig. 1:**
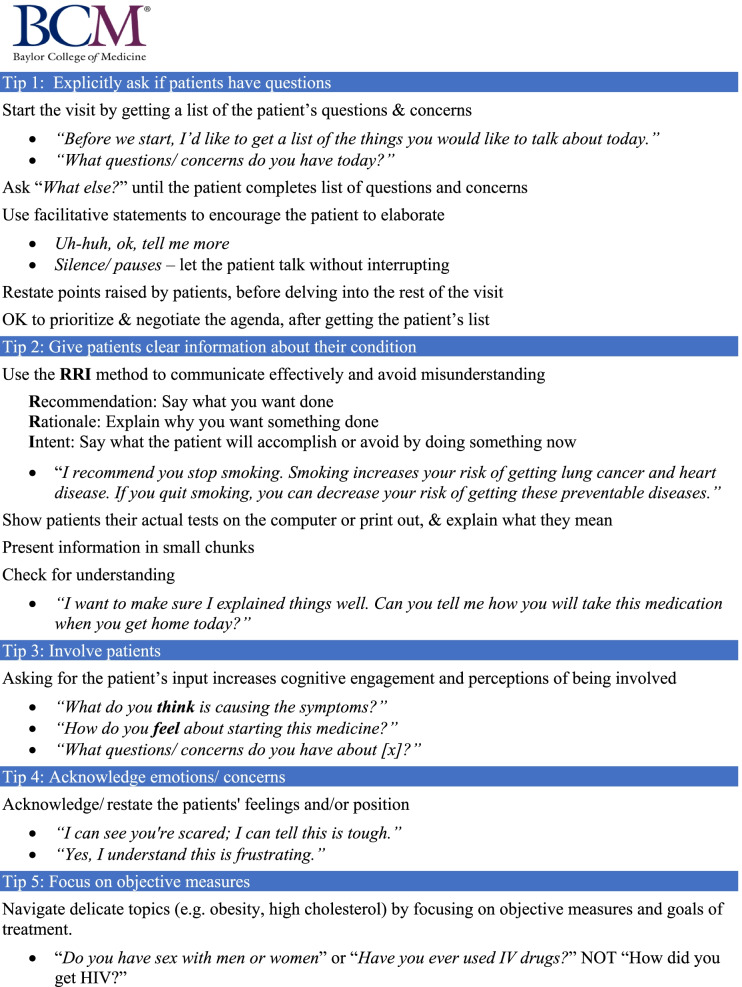
Pocket card given to clinicians taking part in the coaching and feedback program

Once the visit begins, the coach records and watches a live feed of the patient-clinician interaction in a nearby room. The coach takes note of clinician communication behaviors, with attention to strengths and weaknesses within the five targeted behaviors (see Fig. 1). While watching the live feed, the coach also time stamps periods during the visit that illustrate the clinician’s effective use of communication strategies and periods in which more effective behaviors could be used. As soon as the visit ends, the coach uses the time stamps to find and create short video clips showing communication strengths and moments when communication tips can be helpful. Video clips are critical elements of the coaching program. They are used during the feedback session to promote discussion and foster structured self-reflection. The process of making video clips takes about 30 min, and within 30 min of the encounter, the coach is able to meet with the clinician, pending workflow feasibility.

### Video technology

The coaches used Skype for Business 2016 and encrypted laptops to securely live stream and record patient-clinician encounters from a room nearby. Coaches used a hot spot device that provided a stable wireless connection for video recording. Video Studio Pro 2019 editing software was used to create video clips. There were no technical failures or confidentiality issues during filming.

### Feedback sessions

Coaches introduce themselves to the clinician and agree on a time frame for video recording in their clinic. Clinicians receive a pocket card containing communication tips for the five behaviors that are the focus of the coaching program (See Fig. 1). Other than the pocket card, clinicians do not receive guidance or instruction prior to the first coaching session. Coaches consent patients who come in during the selected time frame for the coaching intervention. Clinicians are able to decline recording of visits if they deem the visit not suitable for recording.

Feedback sessions between coach and clinician take about 15 min. The feedback session begins with the coach asking the clinician open-ended questions about his or her goals for communication coaching (e.g., communication areas they want feedback on), as well as the clinician’s thoughts on what they did well and parts of the encounter that they thought were difficult. The coach uses the first few minutes to develop trust and rapport with the clinician and create a safe and supportive environment for receiving feedback. The coach then highlights effective communication strategies used during the visit and identifies areas for improvement.

When delivering feedback, the coach discusses no more than two communication tips. Coaches choose the two tips based on areas in which the clinician needs improvement. The focus on two tips in a coaching session is derived from goal setting literature, which emphasizes that goals are more effective if they are few in number [53]. While discussing the tips, the coach refers to their evaluation notes and shows illustrative video clips from the clinician’s visit. The coach also conducts role play with the clinician so that the clinician can practice the action discussed. At the end of the session, the coach and clinician identify communication tasks the clinician intends to practice (i.e. implementation intention) [54]. Coaches provide a one-page handout summarizing tips discussed during the feedback session.

In this study, Veterans Administration (VA) clinicians received one session of observation and feedback. Clinicians at the VA were the first to participate in the study, and the focus of the coaching intervention at the VA was to assess initial engagement and buy-in. Clinicians at the county clinic received 3–4 sessions, with subsequent feedback sessions focusing on communication tasks discussed during previous coaching sessions and new communication tips. Multiple coaching sessions provided opportunities for clinicians to set goals and continue improving their practice of the communication behaviors taught [55].

### Training coaches

To be effective, feedback must be delivered in a manner which is timely, individualized and non-punitive. Because of the personal and complex nature of communication behaviors, program coaches were carefully trained over 3–4 months to quickly develop client trust and rapport, efficiently observe and rate the five targeted communication tasks; give feedback in a way that is tactful, respectful and psychologically safe; and become proficient in using the technology (e.g., setting up the live feed, making video-clips).

#### Preparing coaches: rigorous interdisciplinary training program

The training for the coaches was developed in conjunction with Lacey Schmidt, PhD, an industrial/organizational psychologist at Minerva Work Solutions, PLLC. Coaches in the intervention were not clinicians, as we wanted to examine the feasibility of using non-clinicians to conduct these sessions. Both coaches in this study have MPH degrees in health promotion and behavioral sciences (Johnson, Njue-Marendes).

### Coaches receive training using the audit and feedback format

Coaches were trained to give feedback by a panel of experts in clinical care (Dang, Giordano), audit and feedback (Hysong, Schmidt), intervention mapping (Markham), and health communication (Street). Prior to training, the research team created a video repository of patient-clinician encounters collected at the research sites. During training, the expert panel met with coaches on a weekly to biweekly basis. Meetings were spent watching encounters from the video repository, and then giving coaches an opportunity to practice observing and rating communication behaviors of interest and providing feedback. Members of the team played the role of the recorded clinician so that the coach could practice delivering feedback on each of the five communication behaviors to clinicians with varying levels of communication skills. All experts evaluated the coaches and gave coaches feedback on the practice session – what went well and what, if anything, they can improve.

Throughout this process, the research team refined and standardized a branching script with feedback language for the coach to use with the provider. For example, scripts that minimize threats to clinicians’ self-esteem include “You’re the medical expert. I’m just the communication coach so that’s what we will be focusing on today” and “Since my background is not in medicine, our coaching session will only focus on communication tips.” Scripts also included empowering language and strategies for engaging clinicians in the coaching sessions, such as asking open-ended questions. Coaches were rated using a performance rubric. Once coaches were confident in basic skills, meetings focused on skillfully working with resistant clinicians, creating psychological safety during feedback sessions, and establishing credibility [48]. After the intervention began, coaches continued to receive feedback on their performance. The principal investigator (Dang) watched live feeds of feedback sessions throughout the study to give same day feedback to the coaches and check for fidelity. Coaches also received continuing one-on-one feedback and training from the expert train-the-trainer coach (Schmidt).

## Results^1^

We successfully deployed this intervention with 23 clinicians at two primary care clinics (15 at the VA and 8 at TSHC). Our participation rate was 76% for clinicians. Lack of time was a typical reason for choosing not to participate. A total of 23 clinicians took part in the coaching intervention. These included 15 internal medicine residents (physicians in training, immediately after medical school), 4 fellows (physicians in post-residency, subspecialty training), 3 attendings (independently practicing physicians) and 1 nurse practitioner. A total of 14 (61%) were male.

Patients and clinicians indicated that the coaching intervention was not burdensome. Patients did not mind the recording; all said that the recording had no impact on their experience and therapeutic relationship with the clinician. Overall, 91% of clinicians would “probably” or “definitely” recommend the coaching program to other clinicians; and 91% reported that the length of the feedback session “was just right.”

### Survey

Responses to the survey indicate high acceptability of the coaching intervention, with high scores across all domains: feedback quality, feedback content, and source credibility. Mean responses ranged from 6.4 to 6.8 out of 7. See Table 3. The high ratings on the survey align with our qualitative results that follow.

**Table 3:** Clinician Feedback Survey Items (*n* = 23)

Items	Mean	Response Values^a^						
		1	2	3	4	5	6	7
		% of clinicians with response values above						
**Feedback Quality**								
The coach gave me useful feedback	6.6					13	13	74
The feedback I received from the coach is helpful	6.5			4		9	17	70
The feedback session was a good use of my time	6.4		4		9	9	13	65
I value the feedback I received from the coach	6.6				4	9	13	74
The feedback I received from the coach helps me communicate more effectively with my patients	6.4					22	17	61
**Feedback Delivery**								
The coach was tactful when giving me feedback	6.7						26	74
The coach made me feel comfortable	6.7						26	74
The coach respected my thoughts and opinions	6.8						22	78
**Source Credibility**								
I respect the coach’s thoughts and opinions about my performance	6.7						30	70
The information I received from the coach was fair	6.7					4	26	70

^a^Responses were on a 7-point scale: 1=strongly disagree, 2=mostly disagree ; 3=somewhat disagree, 4=mixed, 5=somewhat agree, 6=mostly agree, 7=completely agree

### Qualitative interviews

#### Key features of the coaching program

Our analyses of the interviews identified key features of the coaching program, that relate to acceptability and which clinicians said were integral. Key features include: 1) coaches were credible and supportive, 2) feedback was useful, 3) video-clips allowed for self-reflection, 4) getting feedback on the same day was useful, and 5) use of real patients preferred over standardized patients. Each element is detailed below.

1. Coaches were credible and supportive

When clinicians were asked about their impressions, they focused on the coaches’ supportive tone and their credibility.

##### Coaches create a safe environment for feedback

Feedback delivery was the highest rated program characteristic in the survey. Clinicians described coaches’ delivery of feedback as “nonconfrontational,” “nonjudgmental,” “friendly,” and done in “a tactful way.” Clinician 1 said, “She made me feel like it was completely *nonjudgmental* because when I first signed up I thought to myself ‘hmmm how open to feedback am I going to be?’” Clinician 2 said a balance of affirming and constructive feedback made him feel more comfortable, noting that the coach “drew out both things that went well and things to work on, so that I felt good about what I did but also had some good *targetable actions* for the future.”

##### Coaches focus on communication, not medicine

The majority of clinicians described the coaches as a credible source of information and feedback. Because coaches were not clinicians, several participating clinicians thought that the feedback session was less prescriptive, and as Clinician 2 stated, they “didn’t feel intimidated or anything like that.” Clinician 4 said non-clinician coaches were more likely to identify with a patient and focus solely on clinician communication. They had concerns that clinician coaches, in contrast, might be distracted by the medical aspects of an encounter (e.g. diagnostic work-up, treatment decisions). Only one of the 23 clinicians, Clinician 5, found the coaches lacking in credibility because, they said, the appropriate coach is “someone who is doing patient care.”

##### Coaches come prepared

The coaches’ preparation contributed to clinicians’ impressions of their credibility. Clinicians who discussed the credibility of coaches pointed out the importance of coaches coming prepared to feedback sessions. Many found coaches credible because they focused on specific communication tasks and came with prepared video clips. Two clinicians noted that coaches could cite the research supporting the importance of specific communication tasks, as well as audit and feedback principles. Clinician 6 noted that coaches “were well prepared [for] how to coach me…they had a lot of things written down.”

2) Feedback was useful

Several clinicians talked about the lack of feedback specificity in past training. One clinician felt frustrated because they wanted to know specific things they could do to improve. Clinician 7 said, “A lot of [feedback is] based on patient surveys. The biggest complaint patients would have is that their doctor didn’t show enough enthusiasm or care enough.” In their case, they struggled to organize their thoughts and take notes during visits with patients, and at the same time act in a manner that is caring and attentive. They and others said they needed “specific actions to improve patient care.”

Almost all clinicians said the feedback received from the coach was useful and helpful because the feedback focused on concrete communication tasks and the feedback was specific. In fact, Clinician 8 added, “I liked that there were *concrete* things that were picked out that you could see and there were *specific* things she would refer to.”

3) Video-clips allowed for self-reflection

Nearly all participants said that watching video clips during feedback sessions increased self-awareness and self-reflection. Clinician 9 said:

“Just the fact that you’re doing this *self-reflection*, like ‘Oh how am I doing? I’m going to be on video’ …. And then seeing that one little minute clip here…. When are you going to have the opportunity for that?”

Many said the video-clips were the most useful part of the feedback session, and for most, it was the first time they had ever seen themselves talking to a patient. In addition to learning new strategies, clinicians also talked about the video-clips reinforcing desired behaviors. For Clinician 5, the video clip reinforced desired behavior because they could see patients respond positively. They said that “seeing patients appreciate [effective communication and seeing] a benefit” motivated them to continue to use the strategies.

Some clinicians who received more than one coaching session also liked watching video clips in follow-up sessions, showing change in practice. Clinician 6 said:

“That’s what’s sticking to me the most. Going over the [most recent] video and then saying ‘here’s what we saw, here’s what we practiced, here’s what you did. What would you do differently?”

4) Getting feedback on the same day was useful

Clinicians liked getting feedback on the same day. Clinician 3 said the program provided him “the opportunity to get some *real-time feedback* and…see my own interaction from a different person’s [perspective].”

Clinicians also felt that viewing the video clips soon after the patient encounter augmented feedback because their recollection of the situation and context is fresh.

“I could immediately go back, like I remember this interaction. I remember what I was thinking, and I remember, here's how I said that.”

5) Use of real patients was preferred over standardized patients

Clinicians said it was useful to get feedback based on direct observation of encounters with real patients. When asked to compare observations of encounters with standardized versus real patients, clinicians said they prefer the use of real patients. Clinician 4 said:

“The whole standardized patient interaction, the whole time you know it’s all artificial because this person is not a real patient with real symptoms or real problems…. So I think doing that same exercise with *real patients*… [is] more helpful.”

To Clinician 4, interactions with a standardized patient feel artificial, and clinician behaviors with a standardized patient may not reflect how they would act with a real patient.

#### Situational factors

##### Motivation to take part in coaching

When clinicians discussed motives for taking part in the program, a recurrent theme was the desire to be more effective communicators, though reasons for wanting to improve differed. Clinicians framed their motivation as a responsibility to self and patients. Most stressed the importance of effective communication skills, saying effective communication is a “good thing to do” or the “right” thing to do as a clinician, implying a sense of responsibility to their profession. These clinicians were interested in “anything that helps me become a better communicator” (Clinician 2). Some clinicians expressed their sense of responsibility to their patients. Clinician 2 also wanted to improve his own clinical skills so that “there’s nothing [more] I can be doing to [provide] a better experience for the patient.” Clinician 11 focused on their institution’s performance measures and used language common to quality improvement goals and metrics**:** “More effective communication…can help improve patient outcomes and satisfaction.”

#### Comments on program format

##### Brief, same day format effective

Two clinicians (6 and 7) discussed the length of coaching sessions; they thought the length of time was good and that sessions occurring on the same day were “efficient” for the coaching program. Two clinicians (3 and 5) noted that the number of coaching sessions needed could vary by clinician; one argued that 4 sessions might be “more than necessary” for a clinician to implement a skill they master quickly, and another argued that the number of sessions should vary according to the clinician’s workload.

##### Working around the clinician’s schedule is key to uptake

Clinicians talked about the need for any coaching program to be mindful of time pressures, particularly with sick patient encounters or in busy clinics. Clinician 3 pointed out that patients who presented in serious condition limited the clinician’s willingness to participate in coaching for the day because of the stress caused by treating the patient’s more urgent needs. Clinician 1 “had to send [a patient] to the ER, and so the video…I wouldn’t say it inhibited me, but it was just an extra thing.” Clinician 11 echoed this sentiment by pointing out that what works well in a lower volume clinic may not work in a busy clinic.

##### Clinicians want and appreciate strategies to save time

Some clinicians thought that incorporating the tips, such as agenda setting, saved time during visits. Clinician 12 described using agenda setting to keep a new patient visit on track, and “whenever [the new patient] started diverging or going off on tangents to talk about something else, we went back to the list.” Clinician 13 thought that asking open-ended questions at the beginning of the visit saved time by better organizing the encounter, “…. it gave the patient the opportunity to ask all of the questions up front, not to come up with a whole bunch of by the ways.”

On the other hand, other clinicians were concerned that incorporating communication tips would take too much time during a clinic visit. Clinician 14 was skeptical that they would use a technique such as asking open-ended questions: “I don’t think so, just because of time constraints.” Others also cited a lack of time during visits and suggested alternative, time sensitive ways of using agenda setting. Clinician 15 stated, “primary care physicians…are too busy” to use the strategies, but “if we could improve [and for example] patients [could] already have a list of what they want to talk about…maybe it would be a little more attractive for them.” Clinician 4 advocated for “anything that could help outside of the [examination] room.” They suggested that, for example, staff checking patients in at the clinic could ask patients to make a list of questions, and that patients could be asked when scheduling the appointment, while checking in or while waiting in the waiting room.

## Discussion

This study indicates that a real-time video-based coaching intervention targeting clinician communication skills is feasible and acceptable to clinicians. The program had a high participation rate and was deployed with little, if any, interruption to clinic flow. The coaches used the live feed and video splicing technology with ease and seamlessly incorporated it into the development and delivery of feedback.

Quantitative data demonstrate strong acceptability, and qualitative data provide insight into key elements of the coaching program, that clinicians say are integral. Specifically, analyses of clinician interviews revealed the following: 1) coaches were credible and supportive, 2) feedback was useful, 3) video-clips allowed for self-reflection, 4) getting feedback on the same day was useful, and 5) use of real patients preferred over standardized patients.

As with any intervention focused on clinician behavior, attitudes towards learning and being coached that may influence uptake, must be addressed in program development. Many clinicians participated in the program because of a moral and personal commitment to improving their communication skills. These responses are consistent with previous work in which personal improvement and moral goals motivate change in practice [17, 18]. Studies also suggest that clinicians who perceive improvement in their clinical skills, as well as those who perceive their relationships with their patients to be closer report more job satisfaction and less burnout [56, 57]. These potential benefits can be used to encourage clinicians to participate in the intervention.

The coaching program’s focus on specific communication behaviors was accepted positively by clinicians. Many communication interventions focus on broad goals, such as persuading a patient to change their behavior, as opposed to specific communication tasks [58, 59]. This program differs from those interventions in that it focuses on a discrete set of tasks that are concrete, which clinicians overwhelmingly viewed as helpful. Many clinicians responded positively to feedback and indicated that they wanted to continue to develop their skills. Using concrete tasks can engage clinicians who struggle with their communication skills and serve as reinforcement for clinicians who practice these behaviors successfully. As medical training programs make efforts to incorporate communication skill training into their curricula, programs such as coaching and feedback are an important way to continue to build on this training [60, 61].

This pilot involved two non-clinician coaches with extensive training who were generally well received by clinicians. Although training requires intense initial effort, the extensive training the coaches received contributed to their ethos. Clinician responses show that highly trained non-clinician coaches can deliver useful feedback and garner the respect of clinicians. It is important to note that many clinicians thought that non-clinicians were better suited to observe and deliver feedback on their communication skills. It also contributed to feedback delivery that was not threatening and “non-judgmental.” These findings are encouraging, as non-clinician coaches are lower long-term cost and if equally effective, heighten scalability and sustainability. Future implementation and dissemination projects might also consider training peer coaches or patient advocates (e.g., social workers, case managers) to become communication coaches. Future training may be condensed to an intensive course for coaches or may use a “train the trainer” approach, with trained coaches providing audit and feedback training to new coaches.

Clinicians also responded overwhelmingly positively to another distinguishing feature of the program, video feedback. They appreciated it for the same reason it is used in education and sports, to provide a tangible behavioral assessment in real time [62–64]. This underscores the desire of motivated clinicians to hear and see how they communicate. Moreover, training coaches to prepare video clips, akin to a highlight reel in team sports, allowed coaches and clinicians to focus exclusively on specific excerpts of the encounter. Clinicians who received more than one training session, pointed out that they could see themselves improve on the clips. This type of longitudinal audit and feedback is more likely to produce discernable effects on outcomes [65, 66].

Despite the benefits of communication skills training, lack of time is a major challenge to clinician uptake, particularly for clinicians in busy practices. In our study, clinicians indicated that keeping the feedback sessions to 15 min and clinician-centered strategies, such as having the coaches work around the clinician’s schedule, were key to clinician acceptability. In creating similar coaching and feedback programs, attention to clinician concerns and needs is paramount. Future areas of research include possibly leveraging online HIPAA compliant platforms (similar to telemedicine apps) where virtual coaches located anywhere in the world can watch live feeds of encounters and give rapid, same day feedback to the clinician. Such live video feed technology increases the potential reach of any communication intervention and has implications for real-world settings, where local health systems or clinics may not have such support and expertise on site.

Our small sample and qualitative approach capture an initial response to the real-time video-based coaching intervention. The results of this study can be used to inform future implementation studies that measure the feasibility of the intervention rigorously. In the future, large-scale implementation of the coaching intervention will enable an examination of the efficacy of the intervention.

## Conclusions

Programs designed to help clinicians improve their relationships with patients tend to be scarce, time-consuming and unable to garner clinician buy-in. Based on the results of this study, a real-time video-based feedback and coaching program can be perceived as acceptable and feasible by clinicians. Although some elements of the program may be adapted to meet the needs and expectations of individual clinicians, overall, clinicians see video-based coaching sessions as acceptable and feasible. Video-based coaching can help clinicians learn new communication skills, in a way that is clinician-centered, theory-informed, brief (less than 15 min) and timely (same day). Our study demonstrates that real-time coaching, using live feed and video technology, is an acceptable and feasible way of teaching communication skills.

## Data Availability

The de-identified datasets used and/or analyzed during the current study are available from the Principal Investigator (BND) on a case-by-case basis, per written request.

## Abbreviations

ACGME: Accreditation Council for Graduate Medical Education
FIT: Feedback Intervention Theory
VA: Department of Veterans Affairs

## Footnotes

1 Clinicians are not referred to by name in this text. All references to clinician gender were assigned randomly and may or may not refer to the clinician’s gender identity.
